# ECMO-associated nosocomial infections in adults: immunopathogenesis and predictive modeling approaches

**DOI:** 10.3389/fmed.2025.1748154

**Published:** 2026-01-22

**Authors:** Jiaxi Jiang, Yongpo Jiang, Yinghe Xu, Sheng Zhang

**Affiliations:** Taizhou Hospital of Zhejiang Province Affiliated to Wenzhou Medical University, Taizhou, China

**Keywords:** artificial intelligence, biofilm formation, extracorporeal membrane oxygenation, immunopathogenesis, nosocomial infection, predictive modeling

## Abstract

Extracorporeal membrane oxygenation (ECMO) is a critical life-support intervention for patients with severe cardiopulmonary failure. However, its use is associated with a substantially increased risk of nosocomial infections, with reported incidence rates ranging from 8.8% to 64.0%. These infections—particularly ventilator-associated pneumonia and bloodstream infections—are linked to heightened morbidity, prolonged intensive care and hospital stays, and elevated mortality. This review aims to systematically compile Chinese and English literature published between 2018 and 2025, clarify the unique pathophysiological mechanisms of ECMO-related infections, analyze the limitations and breakthroughs of existing prediction models, and explore the potential role of machine learning in developing personalized early warning systems. Additionally, it seeks to establish a clinical decision-making framework for precise prevention and control. We conclude that improving ECMO infection control requires establishing standardized, clinically applicable diagnostic criteria, conducting a multicenter prospective validation study, and developing transparent, AI-enhanced predictive tools to enable real-time infection monitoring and improved patient prognosis during ECMO support.

## 1 Introduction

Extracorporeal membrane oxygenation (ECMO) is an advanced life-support system increasingly used for patients with reversible, severe cardiac or respiratory failure (1). It delivers vital circulatory and respiratory support, allowing for recovery or definitive intervention. Despite notable advancements in ECMO technology, circuit engineering, and patient selection, significant complications persist. Hemorrhage, thrombosis, and nosocomial infections (NIs) remain principal threats to survival in this high-risk group. ECMO-associated infections are among the most prevalent and consequential adverse events. In a recent meta-analysis, the incidence of hospital-acquired infections during ECMO therapy ranges from 8.8% to 64% (2). This reflects significant heterogeneity across studies, caused by differences in ECMO protocols, patient groups, duration of supportive therapy, and definitions of infection. These infections are associated with prolonged mechanical ventilation, extended ICU stays, increased healthcare expenses, and a 32% higher relative mortality rate than non-infected ECMO recipients (2).

Given these challenges, researchers have explored the epidemiology, risk factors, pathophysiology, and prevention of infections in ECMO recipients. However, significant variability exists among studies regarding diagnostic criteria, surveillance intervals, and infection classification, which hampers comparison. This review aims to provide an updated and comprehensive overview of research on ECMO-associated infections. Topics include infection categories, diagnostic standards (CDC, ELSO, and national protocols), immunopathogenesis, risk modeling, advances in artificial intelligence, and infection prevention strategies. The goal is to inform clinical practice and reduce infection-related morbidity and mortality.

## 2 Materials and methods

This study examined the clinical features, diagnosis, genetic mechanisms, and treatment of ECMO-related infections through a thorough review of the literature. Relevant sources were collected from databases including the China National Knowledge Infrastructure (CNKI), Wanfang Data Knowledge Service Platform, and PubMed, using the following keywords: infection, sepsis, bloodstream infections, nosocomial infections, healthcare-associated infection, ventilator-associated pneumonia, extracorporeal membrane oxygenation, ECMO, extracorporeal life support, ECLS, extracorporeal support, machine learning, AI, Prediction Model, with a publication cutoff of October 2025.

Inclusion criteria included peer-reviewed original research and review articles focused on the epidemiology, clinical features, diagnosis, prevention, treatment, or mechanisms of ECMO-associated infections. Studies on non-infectious ECMO-related complications, case reports, small case series, and non-peer-reviewed literature were excluded. Since this review has a descriptive research design, no formal systematic quality assessment or meta-analysis was performed. Study selection and interpretation were based on clinical relevance, methodological strength, and contribution to understanding ECMO-associated infections. We recognize the inherent limitations of descriptive reviews, including potential selection bias and heterogeneity across studies.

## 3 Definition and epidemiology of ECMO-associated infections

Beyond traditional hospital-acquired infections, patients undergoing ECMO face specific infection risks associated with the ECMO device itself, which require separate consideration. Some scholars generally classify any hospital-acquired infection that occurs during ECMO as either ECMO-associated or ECMO-related hospital infection (3, 4). However, in practice, the infections they refer to are mainly common infections seen in intensive care units (e.g., VAP). A recent meta-analysis revealed inconsistencies in how the nosocomial infection time window is defined for patients on ECMO therapy: some studies set it as 12 h, 24 h, or 48 h after intubation, extending to 24 h, 48 h, 72 h, 5 days, 7 days, or 30 days after ECMO removal. Other researchers defined the window solely as the period from ECMO start to discontinuation (2).

**FIGURE 1:**
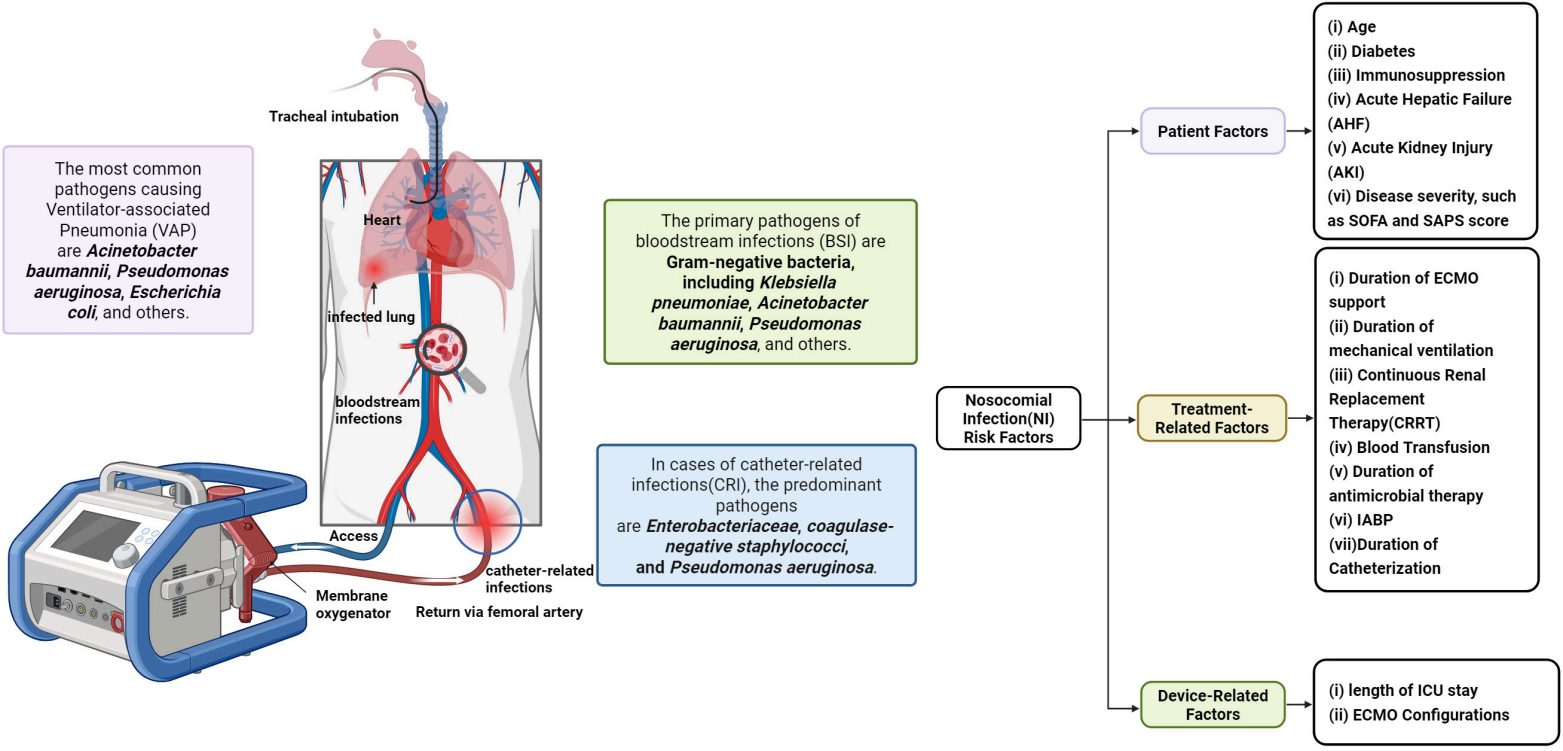
Schematic diagram of ECMO and ECMO-related infections.

The CDC/National Healthcare Safety Network (NHSN) standards are the most widely adopted, offering clear microbiological and clinical guidelines for diagnosing ventilator-associated pneumonia (VAP), bloodstream infections (BSIs), catheter-related infections (CRIs), and catheter-associated urinary tract infections (CAUTIs). VAP and BSI are the most frequently reported (5, 6). In contrast, ELSO guidance highlights standard ICU infection control measures, stringent aseptic procedures during cannulation and circuit management. Cases of primary BSI, which may involve catheter and ECMO-device-related infections, were previously mainly caused by Gram-positive and Candida species (7). In the cases of secondary BSI, mainly due to VAP, Gram-negative species predominated, especially *Acinetobacter baumannii*, *Klebsiella pneumoniae*, and *Pseudomonas aeruginosa* (2). In recent years, the incidence of Gram-negative BSI has risen, and the increased presence of MDR bacteria and fungi (mainly Candida spp.) in ECMO-related nosocomial infection is a concern (8–10). For comprehensive definitions and classifications of infections, see Figure 1.

## 4 Pathogenesis of ECMO

### 4.1 Immune system dysregulation

**FIGURE 2:**
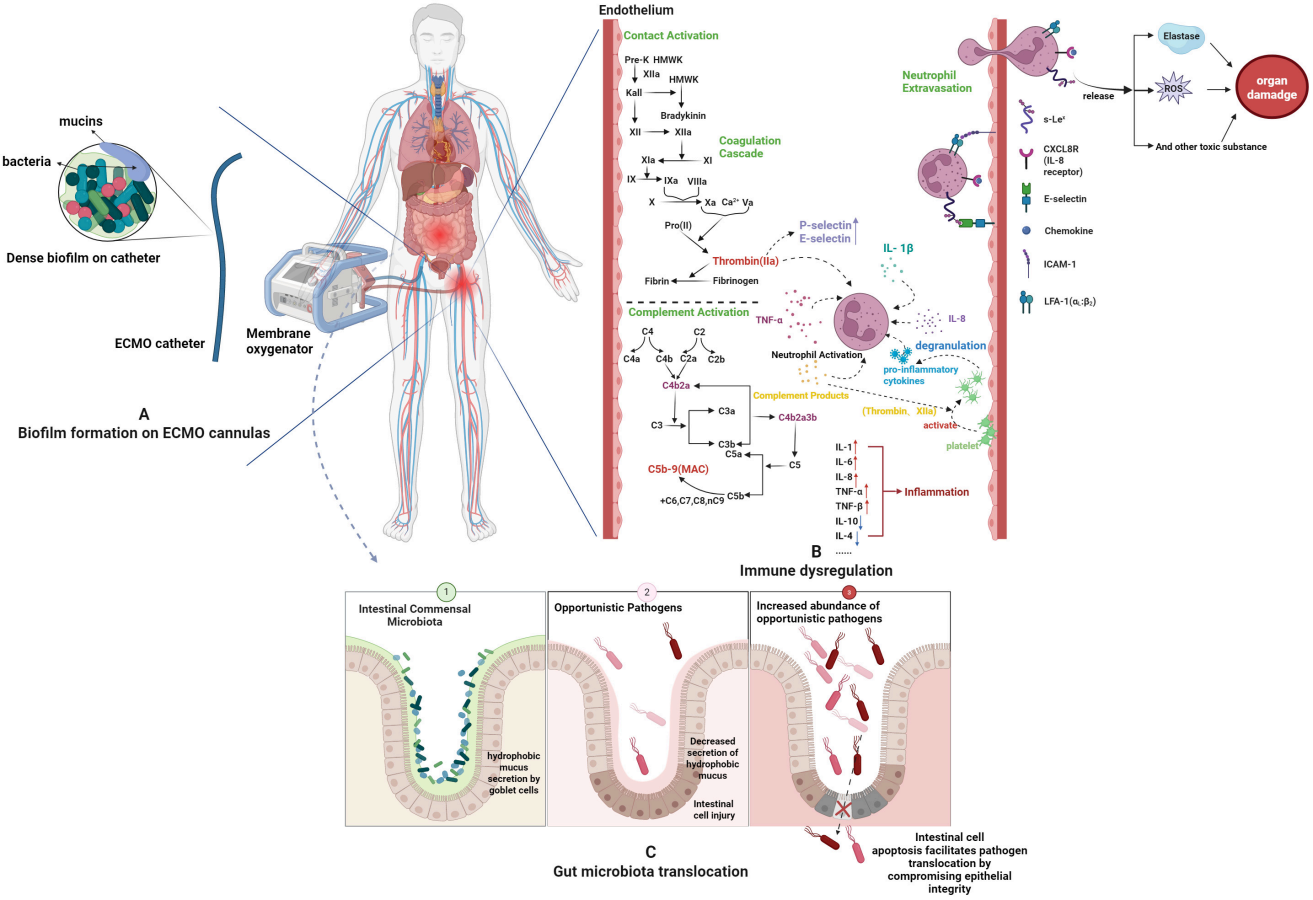
Pathophysiological mechanisms. **(A)** CRAB infection independently predicted the presence of biofilm (OR: 9.60, 95% CI: 2.94–31.30; *P* < 0.001) (23). **(B)** During ECMO, the complement and contact systems are activated due to interactions between blood and biomaterials. The alternative complement pathway (AP) is primarily responsible for generating C3a, C5a, and the membrane attack complex (MAC). This is due to the enhanced hydrolysis of C3 on the biomaterial surface. The contact system drives the production of activated factor XII (FXIIa), which induces the intrinsic coagulation pathway, leading to thrombin formation. Products generated by each of these systems promote the release of proinflammatory cytokines and exert direct effects on leukocytes, platelets, and the vascular endothelium. Specifically, neutrophils are activated, resulting in increased tissue infiltration by neutrophils and ultimately contributing to organ damage. **(C)** The gut lining is shielded by a hydrophobic mucus layer produced by goblet cells, which acts like a defense barrier—blocking digestive enzymes from harming intestinal cells and keeping harmful bacteria and toxins from leaking into the bloodstream. In critically ill patients with insufficient visceral perfusion, however, goblet cells cut back on mucus production, and the mucus loses its water-resistant properties. These patients also develop gut dysbiosis: beneficial gut bacteria (like Firmicutes and Bacteroides) decline, while opportunistic pathogens (such as Proteobacteria) thrive. These leave intestinal cells vulnerable to damage, triggering apoptosis and allowing pathogens to slip into tissues they shouldn’t invade.

Initiating ECMO triggers a systemic inflammatory response similar to systemic inflammatory response syndrome (SIRS) because of direct contact between circulating blood and the non-endothelialized circuit surface (11). This immunological disturbance involves several interlinked pathways (Figure 2).

Notably, most evidence supporting ECMO-induced immune dysregulation comes from small observational studies or extrapolations from extracorporeal circulation models, which limits the ability to identify ECMO-specific immune changes. Additionally, determining the relative contributions of ECMO-related factors versus the underlying critical illness is challenging, exposing significant gaps in research on ECMO-specific immunopathology.

#### 4.1.1 Coagulation-inflammation cross-talk

The cascade starts at the blood–circuit interface, where Factor XII activates the intrinsic coagulation pathway, leading to thrombin production. Thrombin not only converts fibrinogen into fibrin but also increases the expression of P-selectin and E-selectin on endothelial cells, which enhances the adhesion and activation of leukocytes. At the same time, thrombin encourages platelet activation and the release of Platelet-activating factor (PAF) and IL-1β, further boosting cytokine production, including TNF-α and IL-6 (12). Complement activation (e.g., C5a) amplifies this loop by stimulating monocyte tissue factor expression and promoting platelet–leukocyte aggregates, thereby initiating the extrinsic coagulation pathway (13). These mutual reinforcements form a self-sustaining cycle of thromboinflammation.

Although the coagulation-inflammation interaction during ECMO is biologically plausible, direct clinical evidence linking specific coagulation pathways to infection susceptibility remains limited, as most studies focus on inflammatory surrogates rather than infection-related endpoints.

#### 4.1.2 Complement activation and immune suppression

The complement system is one of the earliest innate immune pathways activated during ECMO, with evidence indicating that the alternative pathway is predominant (14). C3a, C5a, and the membrane attack complex (MAC) increase within hours of ECMO initiation. C5a, a powerful chemoattractant, enhances leukocyte recruitment, raises vascular permeability, and boosts the release of proinflammatory mediators. Bergman et al. reported that excessive complement activation during ECMO, especially elevated C5a and terminal complement complex (TCC) levels, impairs leukocyte rheological properties (15). Prolonged and excessive complement activation can exhaust immune supplies, weakening host defenses against pathogens. Complement products, such as C5a, may inhibit neutrophil chemotaxis and phagocytosis when persistently elevated, thus raising the risk of infection (12). This mechanism has been observed in multiple basic studies, but its specific manifestations in clinical practice still require more empirical data support.

#### 4.1.3 Endothelial activation and barrier disruption

Endothelial injury during ECMO occurs in phases. Early complement-mediated activation triggers rapid P-selectin mobilization, promoting leukocyte adhesion and inflammatory signaling (16). Proinflammatory cytokines in the circulation sustain endothelial activation in later phases. TNF-α and IL-1β activate signaling pathways, such as the nuclear factor-kappa B (NF-κB) pathway, in endothelial cells. This leads to continued upregulation of E-selectin, intercellular adhesion molecule-1 (ICAM-1), and vascular cell adhesion molecule-1 (VCAM-1). These changes increase neutrophil rolling, adhesion, and tissue migration (16). Activated neutrophils release elastase and reactive oxygen species, damaging endothelial and parenchymal tissues and contributing to organ injury (17).

#### 4.1.4 Cytokine network imbalance

The initiation of ECMO triggers both proinflammatory and anti-inflammatory cytokine release. IL-6 has dual functions, supporting acute-phase responses and aiding T-cell expansion and B-cell differentiation, while also suppressing other proinflammatory cytokines, such as IL-1β, and increasing the production of anti-inflammatory cytokines, including IL-10 (18). Some studies demonstrate elevated IL-6 levels during ECMO therapy (13, 17, 19, 20). A cooperative study by Risnes et al. showed a negative correlation between IL-6 levels and survival rates, but this pattern was not seen with other cytokines (e.g., IL-1β, IL-8, or IL-10) (13). In this article, IL-6 served as a prognostic marker in these patients. However, due to the small sample size in this study and the unclear role of IL-6 as a pathogenic mediator in ECMO, this research finding cannot be fully confirmed at present. Its universality and clinical predictive value still need to be verified through large-scale prospective studies. Furthermore, IL-8, a powerful activator and chemoattractant for neutrophils, basophils, and T-lymphocytes, is quickly upregulated after ECMO initiation, as confirmed by multiple studies (17). Regarding anti-inflammatory cytokines, a low baseline IL-10 level at the onset of ECMO strongly predicts poor clinical outcomes (21). Most of the above conclusions have been observed in basic research, but their specific manifestations in clinical practice still require more empirical data.

The inflammatory response to ECMO is complex and multifaceted. It remains unclear whether all such excessive inflammatory responses are harmful or could have potential benefits for the host. Based on existing research, in summary, these mechanisms form a “coagulation-inflammation-immunity” triad of vicious cycles: prolonged blood-material contact during ECMO sustains activation of the complement and coagulation systems. When low-dose heparin fails to sufficiently inhibit contact activation, and the endothelial barrier is damaged, a pathological state of hypercoagulability, hyperinflammation, and immunosuppression develops, manifesting as thrombosis, multiorgan dysfunction, and an increased risk of secondary infections.

The establishment of the mentioned mechanism is supported by evidence from animal experiments and simulation models; however, direct validation of its specific effects and underlying mechanisms in clinical populations remains limited. Additionally, there is currently a lack of molecular markers that can specifically distinguish SIRS caused by ECMO from secondary infections. Future research on mechanisms should focus on: (1) Identifying new biomarkers: Search for proteomic, metabolomic, or transcriptomic markers that are uniquely expressed in ECMO patients during infection to replace PCT and CRP, which have traditionally shown limited sensitivity and specificity in critically ill patients. (2) Differentiating ECMO-dependent inflammation: Determine the molecular differences between ECMO-activated non-infectious inflammatory pathways (such as the complement system or coagulation cascade) and those activated by microbial invasion to enable early and accurate diagnosis.

### 4.2 Biofilm formation

Existing studies show that various intravascular devices are susceptible to microbial biofilm formation (Figure 2). These biofilms are surrounded by an extracellular polymeric substance (EPS) matrix, mainly made up of polysaccharides, proteins, and DNA, which microorganisms produce, forming a dense physical barrier (22). Biofilm formation is recognized as a prerequisite for many severe or recurrent infections (23). It is also established as a primary cause of CRI (24, 25).

Based on this, Yeo et al. conducted a study involving 81 ECMO patients with acute cardiopulmonary failure (51 in the infection group and 30 in the control group) (26). Using multimodal detection methods (fluorescence microscopy, confocal laser scanning microscopy, transmission electron microscopy, and semi-quantitative culture), the team assessed how infections affect biofilm formation on ECMO catheter surfaces. They examined clinical factors linked to biofilm development. Results showed a significantly higher biofilm detection rate in the infection group (43.1%) compared to the control group (20%; *p* = 0.034), indicating that infectious conditions encourage biofilm colonization. Multivariate logistic regression identified CRAB infection as the only independent predictor of biofilm presence (OR: 9.60, 95% CI: 2.94–31.30; *p* < 0.001) (26). However, a recent study has revealed that the EPS matrix was also detected on the surfaces of uninfected catheters (27), suggesting that EPS might originate from both microbial and human sources, with human components possibly playing a more crucial role as the foundation for initial microbial adherence and biofilm structure. The deposition of human matrix proteins on medical device surfaces occurs immediately after device implantation. These “conditioning films” originate from microorganisms and the human body, providing a basis for initial microorganism adhesion. Additionally, biofilm formation was found inside the ECMO membrane oxygenator and the ECMO tubing. Diop et al. discovered in a prospective study that biofilm colonization within membrane oxygenators is more common and severe than in intubated patients (28). The study found that, despite the limitations of traditional microbial culture techniques (0% culture positivity rate), scanning electron microscopy (SEM) and 16S rRNA metagenomic analysis detected physically significant biofilms on 100% of the membrane oxygenator surfaces, with bacterial DNA detected in 89% of samples (28). This finding not only confirms that the membrane oxygenator is a critical site for biofilm formation within the ECMO circuit but also alerts clinicians that, even in the absence of positive blood cultures, biofilms in the membrane lung can still elicit systemic inflammatory responses or contribute to bloodstream infections in patients. This has important implications for ECMO infection control strategies.

Biofilms, as dense physical barriers, significantly hinder antibiotic penetration, obstructing drug delivery to deeper layers and impeding sterilization. Additionally, antibiotics can be adsorbed or degraded by EPS during biofilm penetration, leading to a gradual decrease in drug concentration from the outer to inner layers, ultimately preventing the drugs from reaching adequate bactericidal levels (26).

The antibiotic resistance of biofilms results from multiple synergistic mechanisms. First, the physical barrier created by EPS significantly slows down antibiotic penetration. For example, *Pseudomonas aeruginosa* biofilms extend the diffusion time of ciprofloxacin from 40 s to 21 min (29). Second, the slow growth rate of biofilm-associated cells reduces antimicrobial uptake, thereby enhancing resistance (30–32). Additionally, the maturity of biofilms impacts drug susceptibility. Studies demonstrate that mature (10-day-old) *P. aeruginosa* biofilms exhibit 80% higher resistance to tobramycin and piperacillin compared to immature biofilms (33). A survey by Kreitmeier et al. on ECMO catheter colonization further corroborates this finding (34). They observed that approximately 51.7% of patients developed ECMO catheter colonization, and about 50% of colonized catheters yielded positive culture results upon removal, despite patients receiving appropriate antibiotic therapy. Additionally, using anti-Pseudomonas β-lactam antibiotics during catheterization was linked to a higher risk of colonization. However, colonization did not lead to an increased risk of infection or worse prognosis (34).

Although evidence for the prevalence of microbial colonization in ECMO components and its clinical significance is limited, recent studies indicate that we should approach widespread bacterial colonization within the circuit cautiously. For asymptomatic patients, adhering to the principle of on-demand intervention is essential to prevent unnecessary testing or frequent replacement of consumables that could increase patient risk. More importantly, when investigating unexplained sources of infection, the focus should not be limited to the cannulation site. Biofilm colonization within the membrane oxygenator is a critical factor that must not be overlooked. This dual strategy—conservative management of cannulation sites combined with increased awareness of the membrane lung—represents a new approach to ECMO infection control.

### 4.3 Gut barrier disruption and dysbiosis

The gut microbiota plays a pivotal role in human health and disease, with growing recognition as a dynamic “organ” whose dysbiosis is closely linked to organ failure and adverse clinical outcomes (35).

Critically ill patients often show dysbiosis caused by several factors: splanchnic hypoperfusion during shock, systemic inflammation, impaired immunity, dietary changes, pharmacotherapy (such as antibiotics), and decreased intestinal motility. This dysbiosis is characterized by a depletion of beneficial microbes (like Firmicutes and Bacteroidetes) and an overgrowth of harmful bacteria (such as Proteobacteria) (36, 37). Reduced anaerobic populations lower SCFA concentrations, exacerbating epithelial apoptosis, malabsorption, diarrhea, and bacterial translocation (38). Critically, gut dysbiosis has been associated with nosocomial infections, sepsis, multiorgan failure, and even COVID-19 severity (35).

The human intestinal wall is coated with a hydrophobic mucus layer produced by goblet cells, which protects enterocytes from digestive enzymes and prevents the translocation of bacteria and toxins into the bloodstream (37). In critically ill patients with splanchnic hypoperfusion, goblet cells show decreased mucus production and reduced mucus hydrophobicity, resulting in enterocyte damage, increased apoptosis, and pathogen translocation (35, 39).

During ECMO therapy, cytoskeletal contraction of intestinal epithelial cells (IECs) is a key mechanism driving rapid disruption of the intestinal barrier (40). Studies show that tight junctions in IECs are attached to the apical prejunctional actomyosin ring. Contraction of this ring creates mechanical forces that cause plasma membrane retraction, pulling multiple parts of the apical junctional complex into subapical cytoplasmic areas (40). Proinflammatory mediators, such as tumor necrosis factor (TNF), during ECMO are primary triggers for MLC (myosin light chain) phosphorylation and cytoskeletal contraction in IECs. Notably, citrate—through extracellular calcium chelation—can independently cause cytoskeletal contraction and disassembly of tight junctions, highlighting the importance of careful anticoagulant choice (40) (Figure 2).

Emerging evidence indicates that gut injury during ECMO is not just a side effect of systemic illness but also a main cause of systemic inflammation. Early damage to the intestinal barrier may trigger or worsen ECMO-related SIRS and raise the risk of infection (40). Therefore, gut-protective strategies—including enteral nutrition, SCFA supplementation, and microbiome-targeted therapies—should be prioritized from the start of ECMO support.

## 5 Clinical characteristics of ECMO patients

### 5.1 Non-specific manifestations

#### 5.1.1 Fever or abnormal body temperature

(1) Fever: In many infectious diseases, fever is common. However, ECMO patients usually do not develop a fever because heat exchangers often compensate for heat loss to the environment, maintaining normal body temperature during extracorporeal circulation (41). Clinically, doctors usually see many patients with fever after ECMO extubation, and the complex clinical backgrounds of these patients frequently make it challenging to diagnose the cause of the fever (42, 43). Multiple studies have shown that post-ECMO fever is not primarily related to infectious diseases after extubation. Instead, it is often due to aseptic inflammation and, in most cases, is not linked to mortality (42, 44). Maybe it’s because ECMO can trigger SIRS, regardless of the infection’s cause, as blood contact with the ECMO circuit activates the immune and coagulation systems (13). Since temperature is regulated under ECMO, it is possible that fever, as a sign of ECMO-related SIRS, only becomes apparent shortly after explantation, or that a change in the hypothalamic set point through external cooling causes fever (42). The results need further evaluation in larger and prospective multicenter studies.

(2) Hypothermia: It is defined as a core body temperature below 35°C and can occur during severe infections, especially in cases of septic shock.

#### 5.1.2 Hemodynamic instability

(1) Progressive increase in the requirement for vasoactive medications (e.g., norepinephrine) (45).

(2) Unexplained hypotension or arrhythmias (45).

#### 5.1.3 Respiratory system deterioration

Even with sufficient ECMO support, if there is no expected improvement or if the patient’s native lung function worsens (e.g., lung compliance or chest imaging), this should raise suspicion for VAP or catheter-related pneumonia.

#### 5.1.4 Abnormal laboratory indicators

(1) White Blood Cell Count: an elevation exceeding 12 × 10^9^/L, a reduction below 4 × 10^9^/L, or an increase in the proportion of immature granulocytes (left shift).

(2) Inflammatory markers: Procalcitonin (PCT) and C-reactive protein (CRP) are essential references. The key is in the changing trends: a sudden spike after an initial decline following primary treatment strongly indicates infection. Keep in mind that non-infectious factors, such as SIRS, surgery, or thrombosis, can also cause their levels to rise.

(3) Progressive thrombocytopenia may be an early sign of infection-related disseminated intravascular coagulation (DIC). It should be differentiated from heparin-induced thrombocytopenia (HIT) and ECMO-related platelet consumption.

### 5.2 Specific manifestations

#### 5.2.1 Cannulation site infection

(1) Inspection: Redness, swelling, heat, pain, or purulent discharge around the insertion site.

(2) Ultrasound Examination: May reveal hypoechoic areas (abscess formation) in the subcutaneous tunnel or surrounding the vasculature.

#### 5.2.2 Pulmonary infection (VAP or catheter-related pneumonia)

(1) Respiratory Secretions: Notice new purulent sputum or changes in sputum features.

(2) Imaging: Observe new or worsening infiltrates, consolidation, or cavitation on chest X-ray or CT. Note: Lung fields in ECMO patients, especially those on VV-ECMO, can be complex due to underlying conditions and positioning; comparing with previous images is crucial.

#### 5.2.3 Bloodstream infection (BSI)/catheter-related bloodstream infection (CRBSI)

(1) Often presents with the severe systemic signs of infection mentioned above, but lacks a clear localized infectious focus.

(2) A positive blood culture remains the gold standard, especially when the same pathogen is isolated from both the ECMO circuit and peripheral blood simultaneously, with the time to positivity being considerably earlier in the circuit sample than in the peripheral blood sample.

#### 5.2.4 Oxygenator/circuit colonization or infection

(1) Progressive deterioration of oxygenator function: Unexplained continual increase in transmembrane pressure (TMP), indicating thrombosis, with infection playing a significant role.

(2) Plasma Leakage: May be linked to an intensified inflammatory response.

(3) Visible Changes: Observation of abnormal pigmentation, flocculent material, or masses in the membrane, lung, or circuit (potentially indicating bacterial or fungal contamination or biofilm).

#### 5.2.5 Infections at other sites

(1) Urinary Tract Infection: Pyuria, with or without symptoms.

(2) Intra-abdominal Infection: Often observed in post-cardiac surgery patients or those with mesenteric hypoperfusion, presenting as abdominal distension, decreased bowel sounds, or turbid peritoneal drainage fluid.

(3) Mediastinitis/Sternal Infection: Common in post-cardiac surgery VA-ECMO patients, presenting with sternal instability, wound exudate, or retrosternal pain.

## 6 Risk factors and predictive models

### 6.1 Risk factors

The development of ECMO-associated infections is affected by a complex interaction of patient-specific, treatment-related, device-related, and environmental factors. A comprehensive understanding of these factors is crucial for infection prevention and personalized risk assessment.

#### 6.1.1 Patient-related factors

Patient comorbidities and immunological status are key determinants of susceptibility to infection. Conditions such as diabetes mellitus (5, 46), autoimmune disease (5, 47), chronic organ dysfunction (e.g., cardiac, hepatic, or renal failure) (7, 47), and immunocompromised states (e.g., post-transplantation, corticosteroid or immunosuppressant use) (47, 48) have all been independently associated with elevated risk of infection. Furthermore, high scores on severity-of-illness scales—such as the SOFA and SAPS (48)—reflect impaired organ function and have been shown to correlate with the occurrence of infection (49–53).

#### 6.1.2 Treatment-related factors

Several aspects of clinical management contribute significantly to infection development:

- ECMO Duration: Prolonged ECMO support is an independent risk factor, cited across multiple studies (2, 6, 46–48, 50, 52, 54, 55). Studies show a notable rise in infection rates with longer ECMO durations, indicating a two-way relationship: infections extend ECMO dependence, and extended ECMO use increases the risk of infection (5).
- Mechanical Ventilation Time: Extended mechanical ventilation increases infection likelihood (54), as prolonged mechanical ventilation disrupts the respiratory mucosal barrier, impairs mucociliary clearance, and compromises cough reflexes, facilitating pathogen invasion (47).
- Renal Replacement Therapy: The use of continuous renal replacement therapy (CRRT) is an independent risk factor (48), as concurrent ECMO and CRRT support alter antibiotic pharmacokinetics and hemodynamic stability, reducing therapeutic efficacy (47).
- Blood Product Transfusion: Transfusions, especially of packed red blood cells, can have immunomodulatory effects via cytokines and other bioactive substances, which may raise the risk of infection (47, 48).
- Cannulation Technique and Indwelling Time: Cannulation breaches the skin barrier, providing a gateway for microbes. Improper technique or extended catheter dwell time raises the risk of infection, especially with femoral access routes (6). Biofilm development on the cannula and central lines further exacerbates infection risk by protecting pathogens from antibiotics and immune responses (5).

Clinical studies indicate that dual-lumen cervical venoarterial cannulation is associated with lower infection rates than femoral cannulation (56). Percutaneous techniques are also associated with a reduced risk of infection compared to open surgical approaches (5). Early decannulation, when clinically feasible, together with strict local site care (e.g., antiseptic skin cleansing, regular dressing changes, and circuit replacement), is pivotal in mitigating infection risk (57, 58).

#### 6.1.3 Device-associated and environmental factors

- ECMO Mode: Infection risks vary depending on the ECMO configuration. VA-ECMO is generally associated with higher infection risks than VV-ECMO, though conflicting evidence exists (8, 18, 47). For VA-ECMO, infections at the arterial cannulation site may lead to higher rates. In contrast, VV-ECMO patients, who often need extended ECMO support and mechanical ventilation for respiratory failure, face increased risks of VAP, which raises overall infection rates (47). Regardless of the type of ECMO, it remains an independent risk factor for infection (6, 47).
- ICU Environment: Prolonged ICU stay, as an independent risk factor, is associated with increased nosocomial infection risk. Frequent invasive procedures and inconsistent hand hygiene practices among healthcare workers contribute to the transmission of microbes (59). High staff turnover in ICUs may further exacerbate this issue.
- Equipment Contamination: ECMO device components, including water heaters, touchscreen interfaces, and drainage systems, have been shown to harbor pathogens. In a study by Rhee et al., inadequate disinfection of these surfaces was associated with microbial transmission (60). Daily disinfection and strict adherence to hand hygiene protocols are essential countermeasures.

Given the convergence of these findings, developing real-time, individualized infection risk assessment tools remains an urgent clinical priority.

### 6.2 Traditional prediction models

Given the significant burden of nosocomial infections in ECMO-supported patients, early studies have tried to turn known risk factors into quantitative tools using multivariate logistic regression analyses (61–63). These efforts have resulted in foundational risk prediction models.

Despite their promising performance metrics, these models are limited by several factors. First, most studies are based on single-center retrospective datasets with relatively small sample sizes, which restricts their generalizability. Second, the models are static and depend on preselected variables, which may not fully capture the dynamic and non-linear processes behind infection development in ECMO patients. Lastly, they lack real-time adaptability, making them less suitable for integration into electronic health record (EHR)-based early warning systems.

To improve predictive accuracy and clinical relevance, future efforts should aim at integrating multidimensional data—including laboratory, physiological, procedural, and microbiological features—into adaptive, dynamically updating models. These models can be created using machine learning algorithms, which may enable real-time monitoring of infection risk and early intervention strategies in the ECMO setting.

### 6.3 Artificial intelligence (AI)

**FIGURE 3:**
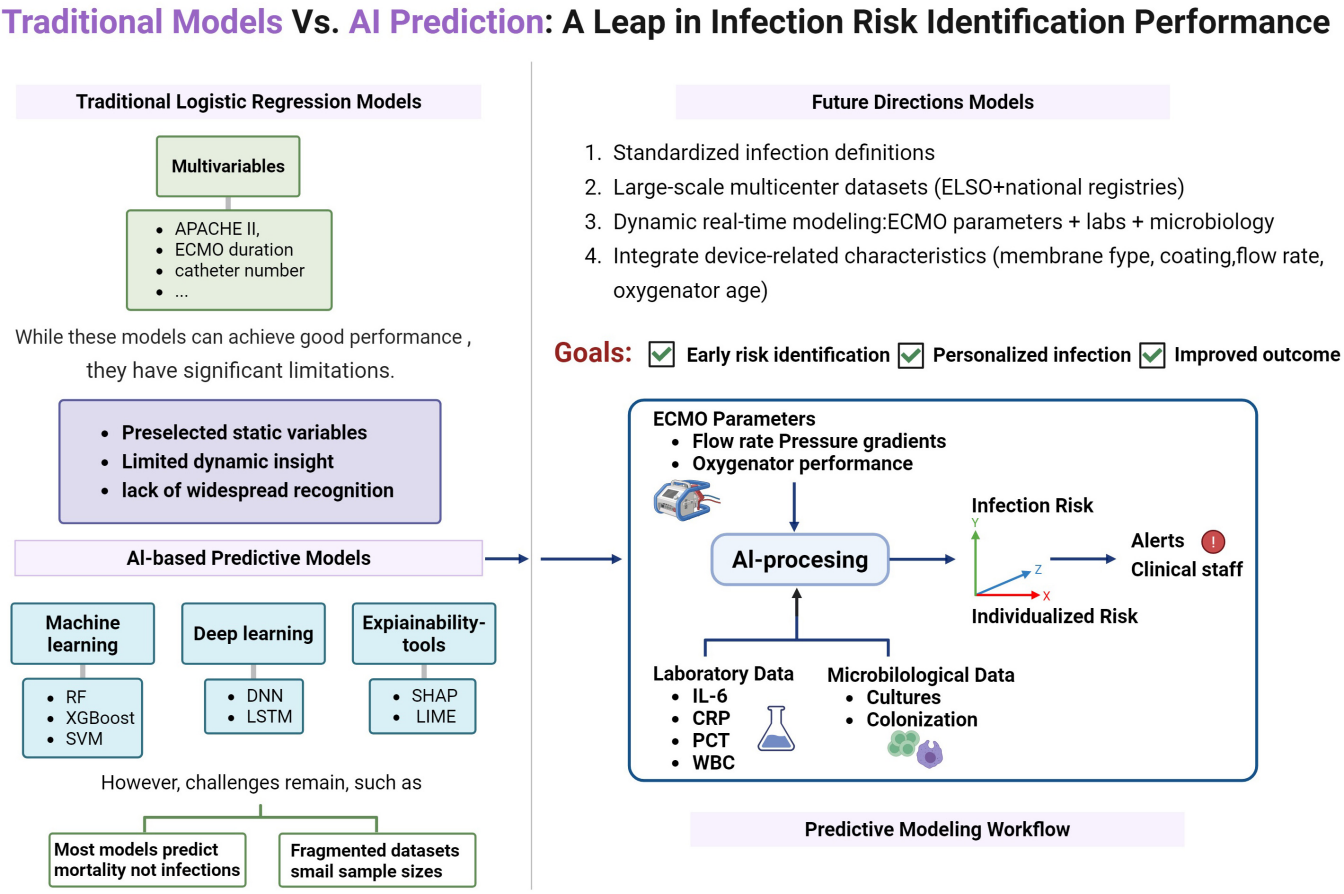
Traditional models vs. AI prediction: a leap in infection risk identification performance.

Artificial intelligence, especially machine learning (ML) and deep learning (DL) techniques, are surpassing the limits of traditional models by incorporating temporal data and multimodal information, showing strong potential to improve infection risk prediction in ECMO-supported patients. Unlike traditional statistical methods, AI algorithms offer greater flexibility and learning ability in handling high-dimensional, non-linear, and multimodal data, including vital signs, laboratory results, real-time device parameters, and unstructured clinical notes (64, 65) (Figure 3).

Artificial intelligence algorithms excel at detecting complex non-linear relationships that traditional regression methods may miss. For example, ML models like Random Forest, Gradient Boosting Machines, and Support Vector Machines have demonstrated higher predictive accuracy in identifying sepsis and septic shock across multiple ICU datasets (66, 67). These models can dynamically incorporate diverse features such as demographic profiles, vital signs, laboratory results, antimicrobial exposure timelines, and ECMO circuit parameters (e.g., flow rate, pressure differentials), continuously updating individualized infection risk scores throughout the ECMO course.

Despite this promise, deploying AI in ECMO contexts poses notable challenges: (1) Small Patient Cohorts and Rare Events: ECMO is a high-acuity, low-volume intervention, resulting in limited patient sample sizes at most institutions. Although the overall infection rate among ECMO patients is substantial, specific infectious events—such as bloodstream infections caused by particular pathogens—are relatively rare within these small cohorts. This rarity poses significant challenges for training statistically reliable ML algorithms, particularly those requiring sufficient event prevalence to avoid overfitting. (2) Non-standardized Infection Definitions: The absence of standardized, consensus-based diagnostic criteria for EAI across healthcare centers represents a significant obstacle. Since ML models rely on labeled outcomes for training, inconsistent definitions of infections (e.g., varying interpretations of VAP) lead to heterogeneous labeling practices. This introduces center-specific biases rather than capturing accurate biological signals, thereby undermining model generalizability and external validity. (3) A key barrier is model interpretability—clinicians must understand and trust the predictions to act on them. Techniques such as SHAP (SHapley Additive exPlanations) and LIME (Local Interpretable Model-Agnostic Explanations), as well as attention-based neural networks, are increasingly being applied to improve explainability and clinician engagement (63, 65). (4) Heterogeneity in Clinical Practice: Substantial variability exists in ECMO management protocols across institutions, including differences in monitoring frequency, weaning strategies, and multidisciplinary team involvement. These inconsistencies lead to non-uniform patterns in clinical data, making it more challenging to develop universally applicable predictive models. (5) ECMO Mode Differences: Predictive models must account for core physiological and technical differences between VA and VV ECMO. These modes support different patient groups, involve separate cannulation techniques, and have varying risks of infection and hemodynamic profiles. Failing to distinguish between modes may hide key risk factors and reduce model accuracy. (6) Underuse of ECMO-Specific Parameters: Current infection prediction models mainly depend on standard ICU variables such as vital signs, lab results, and demographics. Notably, few models include dynamic, ECMO-specific parameters—like pump flow rates, circuit pressure gradients, oxygenator performance trends, and detailed imaging of cannulation sites—although these are relevant to circuit health, biofilm growth, and patient stability. These underutilized data points are valuable sources of information that could improve the accuracy and usefulness of AI-based risk prediction tools. (7) Data Quality and Standardization: The clinical data needed for machine learning—often obtained from electronic health records (EHRs)—frequently suffer from incomplete documentation, inconsistent formatting, and a lack of standardization. Structured data fields containing important physiological or device-related information are often missing, which hampers automated data extraction and limits reproducibility.

In sum, AI represents a promising frontier in ECMO infection risk management. By transcending the limitations of traditional models, AI enables personalized, dynamic surveillance, potentially reducing diagnostic delays, improving outcomes, and guiding targeted infection prevention strategies (68). However, there is no mature model at present.

## 7 Conclusion

Extracorporeal membrane oxygenation serves as a life-saving bridge therapy for patients with severe cardiopulmonary failure; however, its clinical utility is increasingly challenged by ECMO-associated infections—most notably VAP, BSI, and CRI. This review synthesizes the current landscape spanning infection pathogenesis, risk factors, prediction models, and emerging AI-based solutions.

**FIGURE 4:**
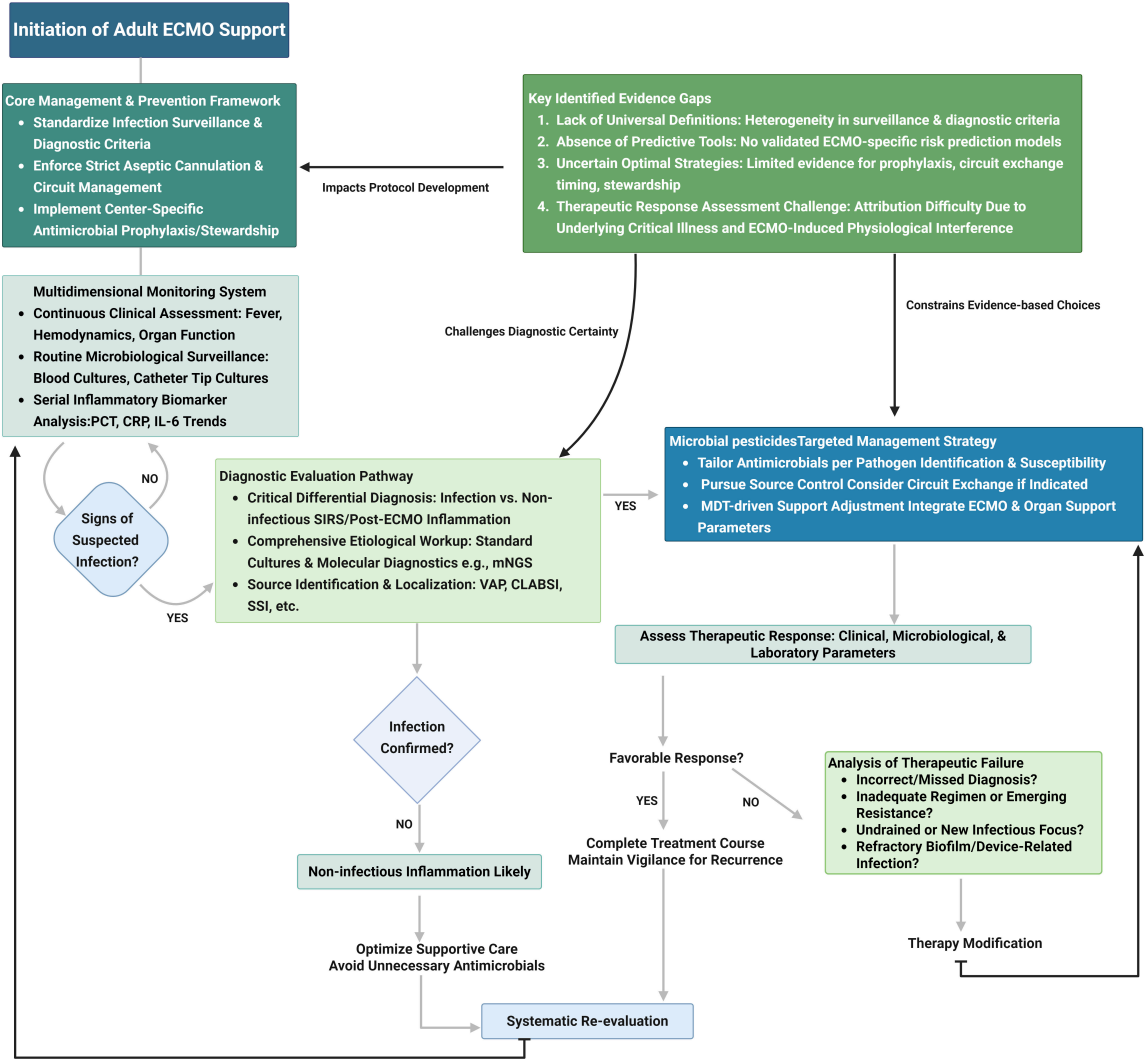
ECMO-associated nosocomial infections: integrated clinical management pathway and evidence gaps.

Despite growing research interest, significant challenges remain (Figure 4). Marked heterogeneity in definitions of infection, diagnostic criteria, and surveillance windows across studies continues to hinder inter-study comparability and the pooling of analyses. Furthermore, most existing predictive models are derived from retrospective, single-center datasets with limited sample sizes, non-uniform variable selection, and a lack of external validation—factors that compromise generalizability and clinical utility.

To address these limitations and enhance infection prevention strategies in ECMO-supported patients, we propose four research priorities:

- Develop standardized diagnostic frameworks and surveillance protocols specific to ECMO-associated infections. Consistency in definitions and timing windows is crucial for accurate comparisons and practical evidence synthesis across studies and registries. For critical care physicians, it is essential to ensure that all ECMO patients undergo consistent physiological monitoring and biochemical assessments, such as oxygenation index, blood gas analysis, electrolytes, and liver and kidney function tests. Additionally, establishing standard operating procedures is crucial to detect complications early.
- Clarify the underlying pathogenic mechanisms, focusing on ECMO-related immune dysregulation, biofilm formation on extracorporeal circuits, and emerging antimicrobial resistance patterns in ECMO-specific contexts. Mechanistic insights will guide targeted preventive and therapeutic strategies.
- Conduct large-scale, multicenter prospective cohort studies to validate infection risk models across diverse patient populations. These studies should aim to quantify the incidence and burden of ECMO-associated infections, refine risk stratification strategies, and evaluate the clinical impact of infection-specific interventions. Hospitals should actively participate in multi-center registration databases or related clinical research networks, share patient data, and promote large-scale multi-center clinical research.
- Intensive care physicians can utilize interpretable machine learning algorithms to combine multimodal data—such as real-time ECMO parameters (e.g., flow rates, pressure gradients), serial immunoinflammatory biomarkers, and gut microbiome signatures—to monitor individual infection risks, support early clinical decision-making dynamically, and create an AI feedback system to regularly assess AI performance. Adjust the algorithm or clinical strategy based on these outcomes.

By addressing these gaps, future research can pave the way for precision infection surveillance frameworks and evidence-based interventions, ultimately improving patient safety and outcomes during ECMO support.

## Data Availability

The original contributions presented in the study are included in the article/supplementary material, further inquiries can be directed to the corresponding author.
